# Evaluation of wing geometric morphometrics for species discrimination of *Haematopota* (Diptera: Tabanidae) in Western Thailand

**DOI:** 10.1016/j.crpvbd.2026.100447

**Published:** 2026-10-09

**Authors:** Pichayapa Som-udorn, Pattarakorn Khongtaweboon, Ayarin Wijitthavornkun, Sivapong Sungpradit, Tanawat Chaiphongpachara, Tanasak Changbunjong

**Affiliations:** a Faculty of Veterinary Science, Mahidol University, Nakhon Pathom, 73170, Thailand; b Department of Pre-Clinic and Applied Animal Science, Faculty of Veterinary Science, Mahidol University, Nakhon Pathom, 73170, Thailand; c The Monitoring and Surveillance Center for Zoonotic Diseases in Wildlife and Exotic Animals (MoZWE), Faculty of Veterinary Science, Mahidol University, Nakhon Pathom, 73170, Thailand; d Department of Public Health and Health Promotion, College of Allied Health Sciences, Suan Sunandha Rajabhat University, Samut Songkhram, 75000, Thailand

**Keywords:** Clegs, DNA barcode, Geometric morphometrics, Landmark, Outline, Wing shape

## Abstract

*Haematopota* (Diptera: Tabanidae) comprises hematophagous flies of veterinary and medical importance, making reliable species discrimination important for understanding their diversity and potential significance. Quantitative wing morphology offers an opportunity to improve species differentiation. This study evaluated the utility of wing geometric morphometrics (GM) for distinguishing *Haematopota* specimens collected in Western Thailand. Four species, *H. javana*, *H. libera*, *H. pachycera*, and *H. varifrons*, were examined using two complementary wing GM approaches based on landmarks and wing outlines. DNA barcoding of the mitochondrial cytochrome *c* oxidase subunit 1 (*cox*1) gene provided complementary molecular support for the morphological species assignments, although no matching reference sequences were available for *H. varifrons*. Wing shape demonstrated greater discriminatory power than wing size in both approaches. Landmark-based GM correctly classified all specimens, achieving 100% classification accuracy in leave-one-out cross-validation. Outline-based GM also performed well, with 97.78% classification accuracy. Landmark-based GM showed higher classification accuracy than outline-based GM, while outline-based GM offered a useful alternative when homologous landmarks could not be reliably digitized. The complementary use of DNA barcoding and wing GM may facilitate species discrimination in *Haematopota* and provide a basis for future studies incorporating broader geographic and population sampling.

## Highlights

• Wing geometric morphometrics (GM) effectively discriminated four species of *Haematopota.*
• Landmark-based GM achieved 100% classification accuracy by leave-one-out cross-validation.
• DNA barcoding provided complementary molecular support for species identification.

## Graphical abstract

**Figure:**
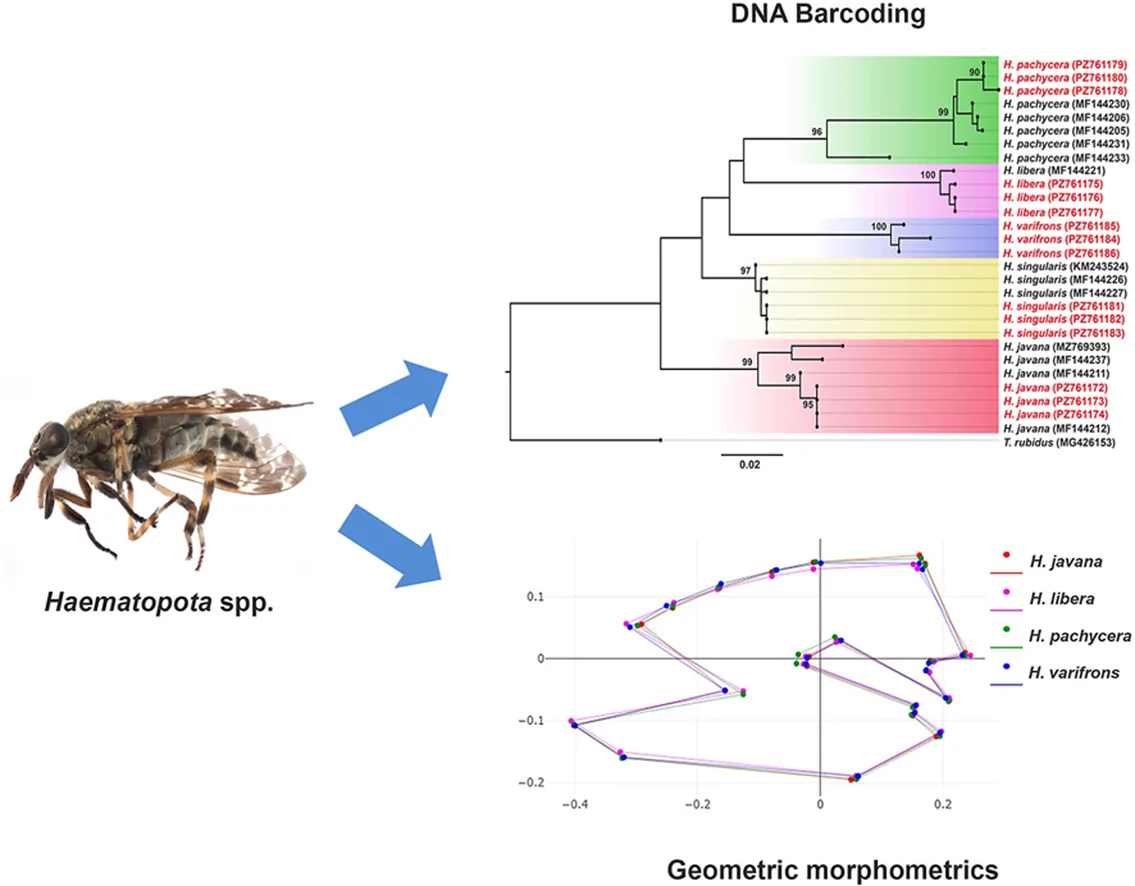

## 1 Introduction

Species of the genus *Haematopota* Meigen, 1803 (Diptera: Tabanidae), commonly known as clegs, are hematophagous insects of considerable veterinary and medical importance. The genus includes approximately 560 described species worldwide, making it one of the most species-rich genera within the family Tabanidae (Bánki et al., 2026). Adult females are obligate blood-feeders that feed on a wide range of vertebrate hosts, including cattle, horses, buffaloes, other wildlife, and humans (Stone and Philip, 1974; Rovie-Ryan et al., 2013; Baldacchino et al., 2014; Mullens, 2019; Bartosik et al., 2026). Their painful bites cause irritation, blood loss, stress, and skin lesions, reducing feeding efficiency and livestock productivity. Heavy infestations can reduce milk production in dairy cattle because the animals spend more time avoiding flies than feeding (Phasuk et al., 2011). Besides their nuisance, species of *Haematopota* are mechanical vectors of several pathogens of veterinary significance, including bovine viral diarrhea virus (Tarry et al., 1991), lumpy skin disease virus (Sohier et al., 2019), *Bacillus anthracis*, *Brucella* spp., *Trypanosoma evansi*, and *Trypanosoma theileri* (Baldacchino et al., 2014). Thus, accurate identification of *Haematopota* spp. is crucial for understanding their ecology, geographical distribution, potential role in pathogen transmission, and impact on livestock health and productivity.

Thailand’s tropical climate provides highly favorable environmental conditions for tabanid flies, given its high temperatures and constant humidity year-round (Phasuk et al., 2011; Changbunjong et al., 2018). These conditions favor the breeding of multiple generations annually and sustain abundant populations across a wide range of habitats. Thailand harbors considerable *Haematopota* spp. diversity, with approximately 40 species reported from Thailand (Stone and Philip, 1974). However, the number of *Haematopota* species recorded from individual regions may be considerably lower; for example, five species were reported from Central Thailand by Phasuk et al. (2011). The western region of Thailand, particularly Kanchanaburi Province, features extensive forested landscapes, abundant wildlife, and diverse livestock production systems that create suitable environments for diverse tabanid species. Previous surveys show that Western Thailand harbors high horse fly diversity (Changbunjong et al., 2025), suggesting the region’s potential importance for *Haematopota* spp. diversity. However, detailed information on *Haematopota* spp. diversity and species distribution in Western Thailand is limited.

Accurate identification of *Haematopota* species using traditional taxonomic methods is often challenging because many species exhibit highly similar external morphology, such as in head morphology, antennal structures, leg coloration, thoracic patterns, and wing patterns (Stone and Philip, 1974). Species-level differentiation often requires careful examination of multiple morphological characters rather than reliance on a single external feature. Many of these diagnostic characters require considerable taxonomic expertise and careful examination of specimens, which may have been altered by preservation or physical damage. Consequently, morphological identification alone may be difficult, particularly for closely related species or specimens with incomplete or deteriorated diagnostic characters, emphasizing the need for integrative approaches to enhance the accuracy of species identification.

DNA barcoding is one of the most widely used molecular approaches for insect species identification and biodiversity studies. The mitochondrial cytochrome *c* oxidase subunit 1 (*cox*1) gene has proven particularly valuable, functioning as a standardized genetic marker that facilitates reliable species discrimination across diverse insect groups (Hebert et al., 2003). Unlike traditional morphological identification, DNA barcoding relies on DNA sequence information rather than external morphological characters and can facilitate species identification even when specimens are damaged, immature, or morphologically cryptic, provided that suitable reference sequences are available. Previous studies have demonstrated the effectiveness of *cox*1 barcoding for identifying tabanid flies, including *Haematopota* spp. and *Tabanus* spp. from Thailand (Nitiyamatawat et al., 2017; Changbunjong et al., 2025), confirming that molecular data can establish species boundaries that are not always evident from morphology alone. Nevertheless, molecular techniques require specialized laboratory facilities and relatively high analytical costs, making them less suitable for routine large-scale surveillance when used as the sole identification method. Therefore, integrating molecular data with complementary morphological approaches provides a more robust framework for species discrimination and identification.

Wing geometric morphometrics (GM) has emerged as a rapid, inexpensive, and reliable method for quantitative species discrimination in insects of medical and veterinary importance (Dujardin, 2008; Wilke et al., 2016; Changbunjong et al., 2023; Hadj-Henni et al., 2023). By analyzing variations in wing size and shape, GM captures subtle morphological differences that are often undetectable using conventional qualitative characters. In particular, landmark-based GM uses homologous anatomical landmarks to quantify wing morphological variations, whereas outline-based GM characterizes the contour of a defined wing region (Dujardin and Dujardin, 2019). Both methods have successfully distinguished closely related species in numerous insect groups, including biting midges (Diptera: Ceratopogonidae) (Hadj-Henni et al., 2023), *Stomoxys* flies (Diptera: Muscidae) (Changbunjong et al., 2023; Weluwanarak et al., 2024), *Tabanus* spp. (Diptera: Tabanidae) (Changbunjong et al., 2024), and mosquitoes (Diptera: Culicidae) (Laojun et al., 2023, 2024; Alvial et al., 2024). Recent studies have shown that integrating GM with DNA barcoding substantially improves species discrimination and taxonomic resolution compared with either method alone (Changbunjong et al., 2025). Despite the successful application of GM in several insects of medical and veterinary significance, its utility for species discrimination in *Haematopota* has not been investigated. This knowledge gap highlights the need to determine whether wing morphological variations provide reliable diagnostic characters for distinguishing among *Haematopota* species.

Given the limited information available on the species composition of *Haematopota* and the lack of quantitative wing-based species discrimination approaches in Western Thailand, this study used mitochondrial *cox*1 DNA barcoding to provide molecular support for morphological species assignments and evaluated the discriminatory performance of wing GM based on wing size and shape using landmark- and outline-based GM approaches. The findings provide baseline data demonstrating the utility of wing morphology for species discrimination in *Haematopota* and contribute to future taxonomic, biodiversity, and surveillance studies in Thailand.

## 2 Materials and methods

### 2.1 Collection sites

Adult female *Haematopota* spp. were collected from beef cattle farms in Thailand in October-December 2025. The main sampling sites were located in Thong Pha Phum and Mueang Districts, Kanchanaburi Province, Western Thailand (Fig. 1A). *Haematopota javana* was collected from Thong Pha Phum District, whereas *H. libera*, *H. pachycera*, and *H. varifrons* were collected from Mueang District. Detailed information on the collection sites, geographical coordinates, and numbers of wing images used in the landmark- and outline-based GM analyses is provided in Table 1.

**Fig. 1:**
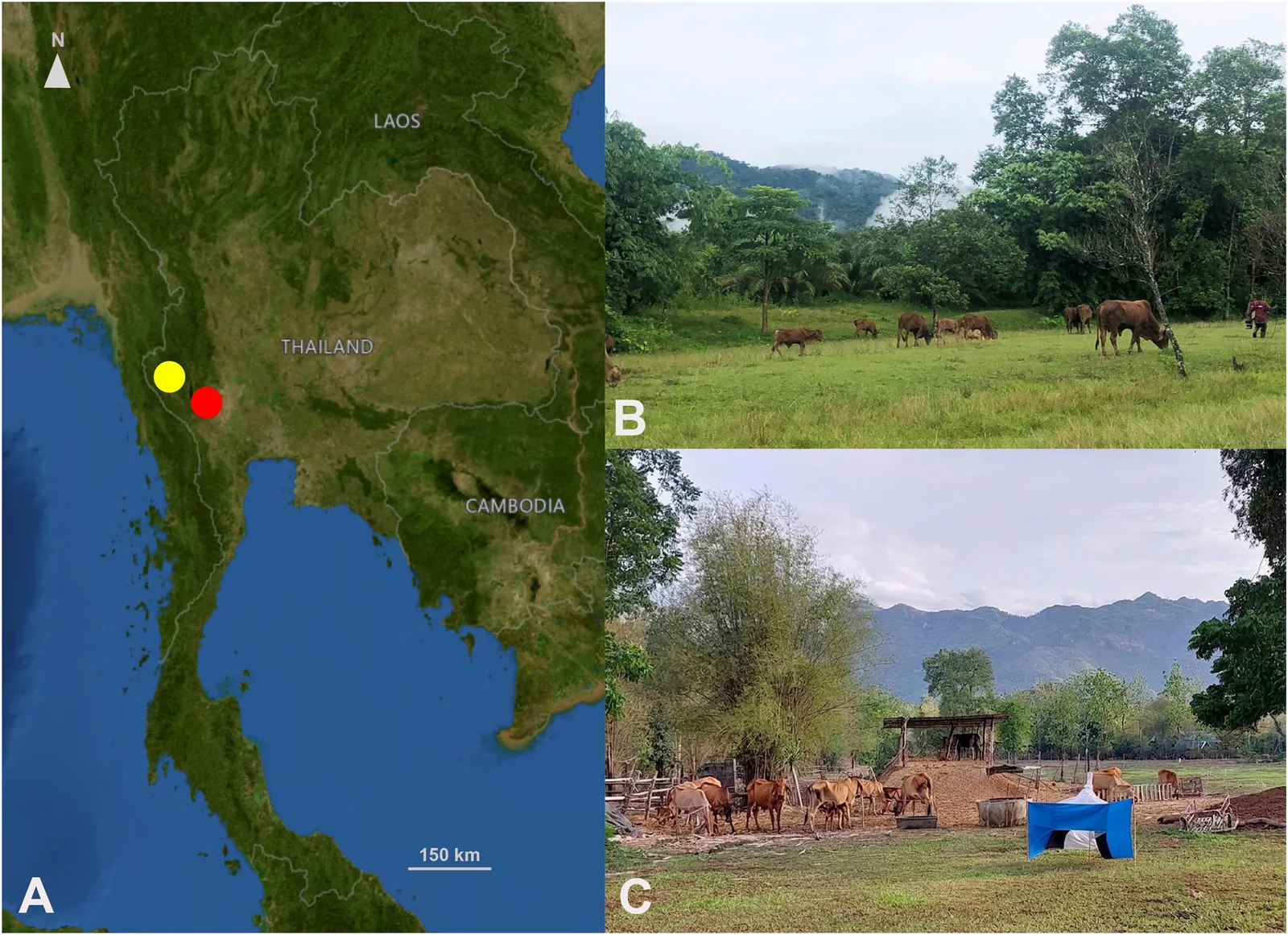
**A** Collection sites in Western Thailand: Thong Pha Phum District (*yellow*) and Mueang District (*red*) in Kanchanaburi Province, Western Thailand. **B** Beef cattle, common hosts of *Haematopota* species in the study areas. **C** Nzi trap used to collect *Haematopota* flies. Map source: USGS National Map Viewer (https://apps.nationalmap.gov/viewer/).

**Table 1:** Collection sites and number of wing images of *Haematopota* species used in landmark- and outline-based geometric morphometric analyses.

Species	District/Province	Coordinates	No. of wing images used	
			Landmark-based GM analysis	Outline-based GM analysis
*H. javana*	Thong Pha Phum, Kanchanaburi	14°39′28″N, 98°32′19″E	45	45
*H. libera*	Mueang, Kanchanaburi	14°11′47″N, 99°15′55″E	45	45
*H. pachycera*	Mueang, Kanchanaburi	14°11′47″N, 99°15′55″E	45	45
*H. varifrons*	Mueang, Kanchanaburi	14°11′47″N, 99°15′55″E	45	45
Total			180	180

### 2.2 Specimen collection and morphological identification

Specimens were collected using Nzi traps (Mihok, 2002) placed near beef cattle or cattle-holding areas to target host-seeking flies (Fig. 1B and C). The traps were operated daily from 6:00 to 18:00 h for 3 consecutive days at each sampling site per month. Specimens were removed from the traps at 2–3-h intervals, euthanized by freezing at −20 °C, and individually stored in 1.5-ml microcentrifuge tubes. After each collection period, specimens were transported to the Vector-Borne Diseases Research Unit, Faculty of Veterinary Science, Mahidol University, for subsequent morphological identification and processing.

Specimens were initially examined based on external morphological characteristics (Fig. 2, Fig. 3, Fig. 4) using the taxonomic key developed by Stone and Philip (1974) under a Nikon SMZ745 stereomicroscope (Nikon Corporation, Tokyo, Japan). Three representative specimens of each morphologically identified species were randomly selected for molecular confirmation by DNA barcoding.

**Fig. 2:**
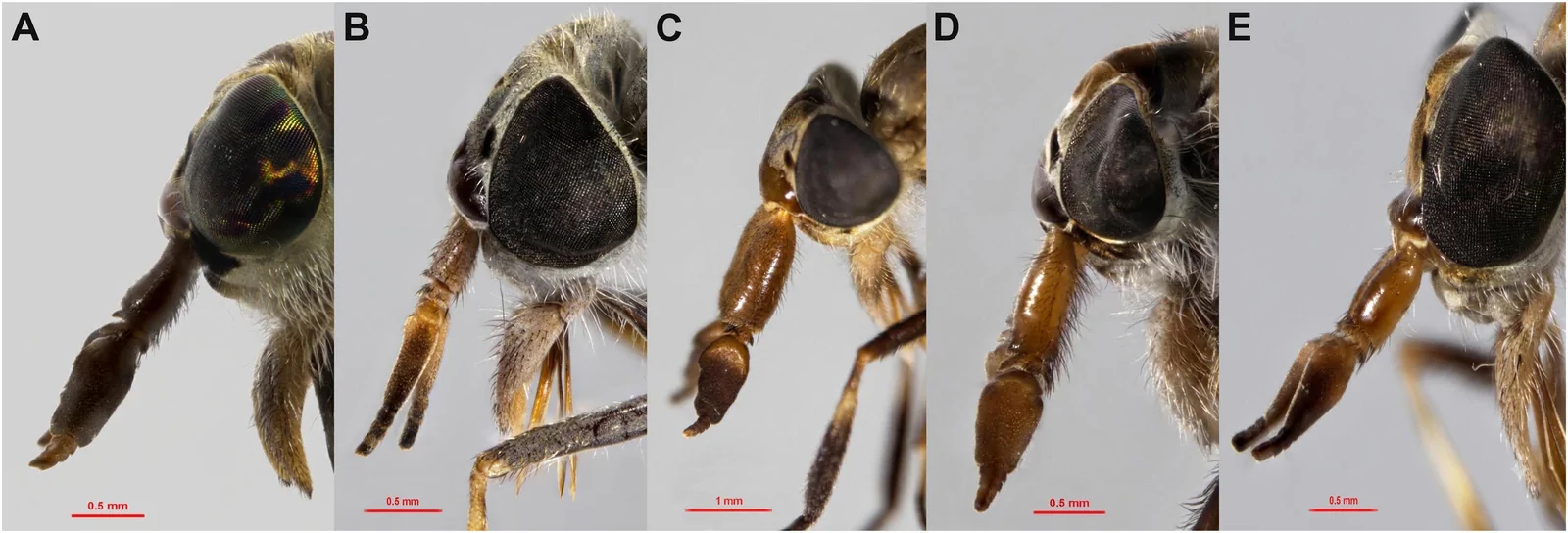
Antennae from five *Haematopota* species: *H. javana* (**A**), *H. libera* (**B**), *H. pachycera* (**C**), *H. singularis* (**D**), and *H. varifrons* (**E**).

**Fig. 3:**
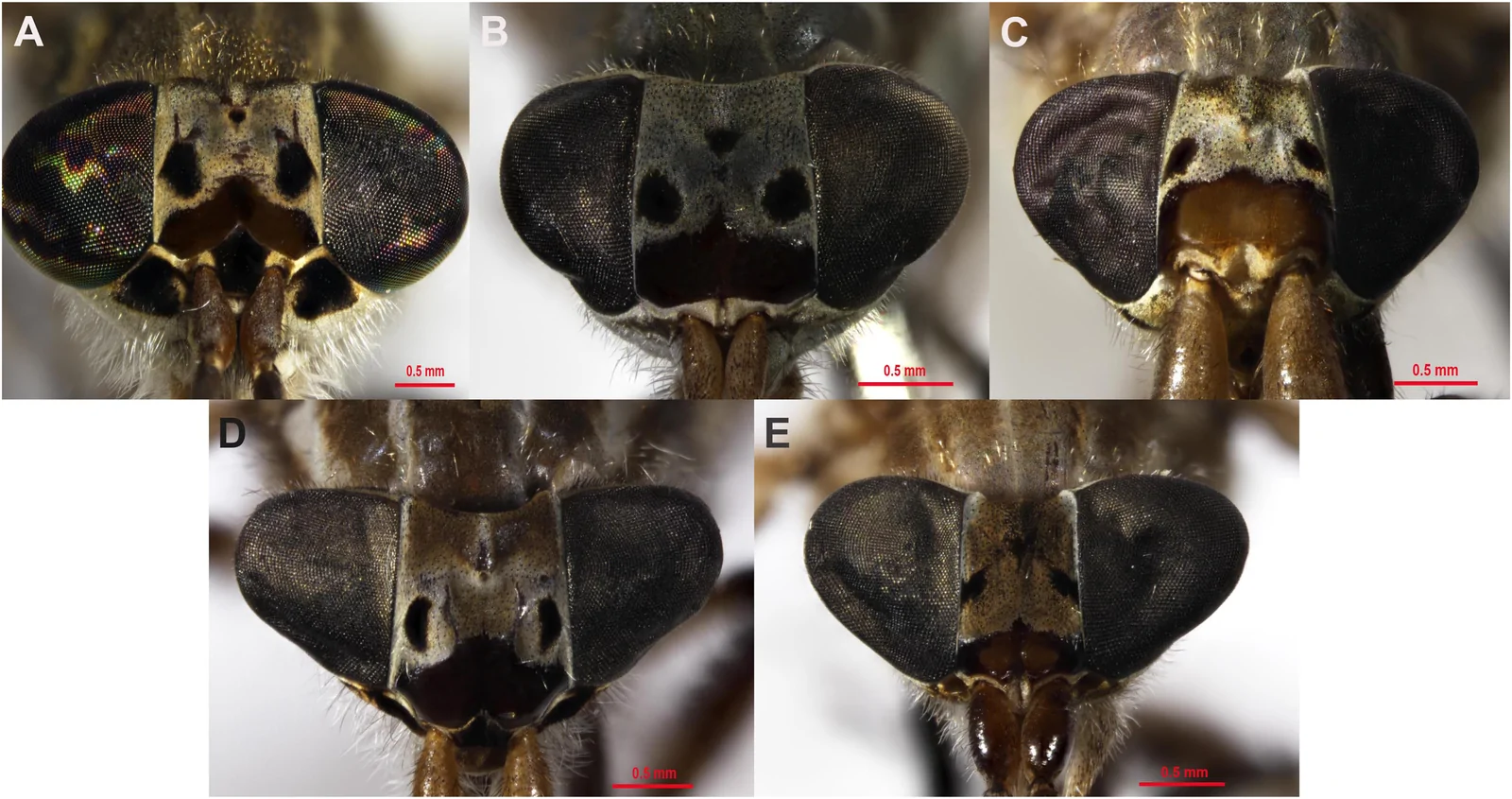
Head morphology of five *Haematopota* species: *H. javana* (**A**), *H. libera* (**B**), *H. pachycera* (**C**), *H. singularis* (**D**), and *H. varifrons* (**E**).

**Fig. 4:**
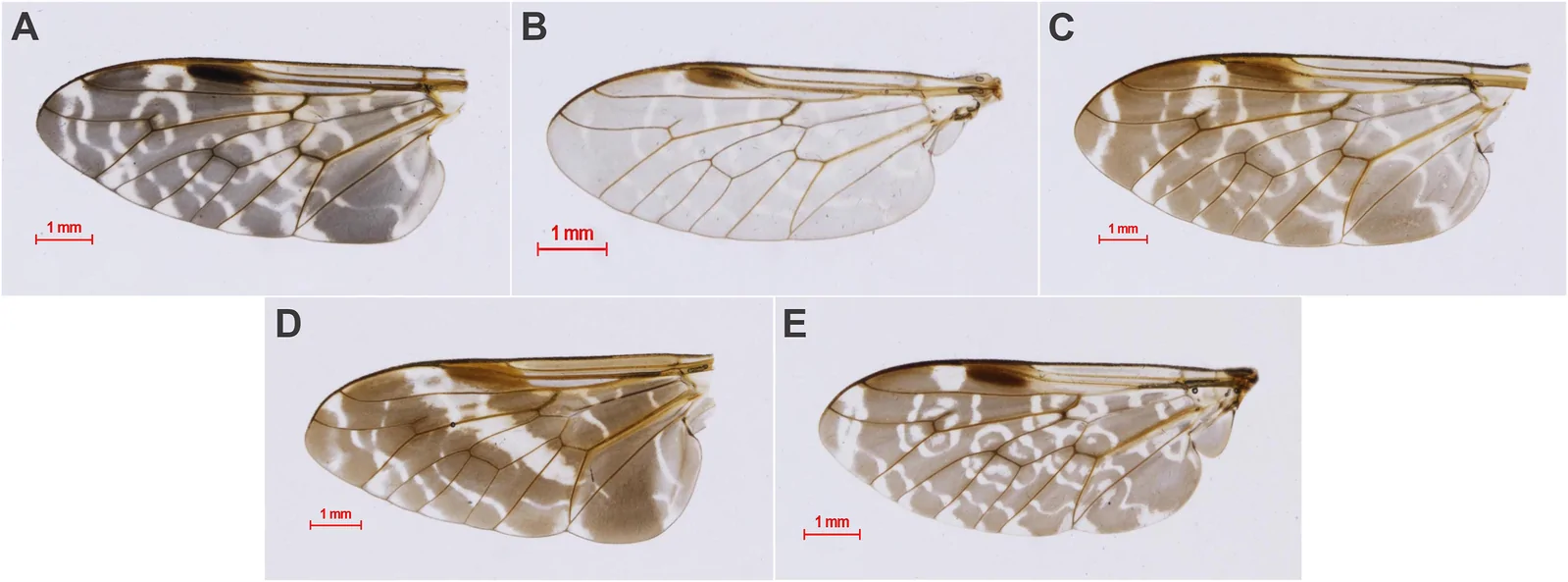
Wing pattern of five *Haematopota* species: *H. javana* (**A**), *H. libera* (**B**), *H. pachycera* (**C**), *H. singularis* (**D**), and *H. varifrons* (**E**).

Four species, *H. javana*, *H. libera*, *H. pachycera*, and *H. varifrons*, were included in the GM analyses. For each species, 45 female specimens were included, and only the left wing was used, resulting in 180 wing images for both landmark- and outline-based GM analyses (Table 1). *Haematopota singularis* was also collected from Mueang District, Kanchanaburi Province, but was not included in the GM analyses because only 10 adult female specimens were available.

### 2.3 DNA extraction, polymerase chain reaction, and sequencing

To confirm the taxonomic identity of specimens prior to GM analysis, DNA barcoding was performed on three randomly selected specimens of each morphologically identified species, including *H. singularis*. Two to three legs were removed from each specimen under a stereomicroscope. Total genomic DNA was extracted using the DNeasy Blood & Tissue Kit (Qiagen, Hilden, Germany) according to the manufacturer’s instructions. *cox*1 was amplified using the universal primers LepF1 and LepR1 (Hebert et al., 2004), targeting an approximately 658-bp fragment.

Polymerase chain reaction (PCR) amplification was performed in a 20-μl reaction mixture containing 1 μl template DNA (approximately 50–100 ng), 0.25 μM of each primer, 1× AllTaq™ PCR Master Mix (Qiagen, Hilden, Germany), and nuclease-free water to the final volume. Amplification was performed with an initial denaturation at 94 °C for 1 min, followed by 5 low-stringency cycles of 94 °C for 30 s, 45 °C for 40 s, and 72 °C for 1 min; 35 standard cycles of 94 °C for 30 s, 55 °C for 40 s, and 72 °C for 1 min; and a final extension step at 72 °C for 10 min (Changbunjong et al., 2025). Each PCR run included a positive control (*H*. *pachycera* DNA) and a negative control (nuclease-free water) to verify amplification and monitor for contamination, respectively.

PCR products were analyzed by electrophoresis on 1.5% (w/v) agarose gels containing SafeView™ FireRed nucleic acid stain (Applied Biological Materials Inc., Richmond, BC, Canada) and visualized using an Azure c150 UV transilluminator (Azure Biosystems, Dublin, CA, USA). Amplicons of the expected size were excised from the agarose gel, purified, and submitted for bidirectional Sanger sequencing to U2Bio (Thailand) Co., Ltd. (Bangkok, Thailand).

### 2.4 DNA sequence analysis

Forward and reverse *cox*1 sequences were assembled to generate consensus sequences, and sequence alignment was performed using MEGA 11 (Tamura et al., 2021). The resulting consensus sequences were used for species identification by comparing them with publicly available sequences in the NCBI GenBank database using the Basic Local Alignment Search Tool (BLAST) (https://blast.ncbi.nlm.nih.gov/Blast.cgi) and the Barcode of Life Data Systems (BOLD) using the Barcode Identification Engine (Barcode ID) (https://id.boldsystems.org/).

Species identities were confirmed by comparing consensus sequences against reference sequences available in GenBank and BOLD. Top-matching sequences were verified to ensure that species assignments were supported by multiple reference sequences, rather than by a single database record. Following confirmation, 15 *cox*1 sequences representing the five morphologically identified *Haematopota* species were deposited in GenBank under the accession numbers PZ761172-PZ761186.

To further validate species identification and explore genetic relationships among the analyzed taxa, maximum-likelihood (ML) phylogenetic analysis was performed using MEGA 11 (Tamura et al., 2021). The Tamura 3-parameter model with gamma-distributed rate variation (T92+G) was selected as the best-fit nucleotide substitution model for the dataset. Nodal support was assessed using 1000 bootstrap replicates. Reference *Haematopota* spp. sequences retrieved from GenBank were used along with the sequences generated in this study for comparative and phylogenetic validation of species identification. *Tabanus rubidus* (Diptera: Tabanidae) (GenBank: MG426153), a member of the suborder Brachycera, was used as the outgroup. The resulting phylogenetic tree was edited and visualized using FigTree v.1.4.4 (Rambaut, 2018).

### 2.5 Wing preparation and image acquisition

The left wing of each selected specimen was carefully detached using a sterile blade. Only intact wings without visible damage, tears, or missing portions affecting the landmark or outline regions of interest were used for GM analyses. Each wing was mounted on a glass microscope slide using Hoyer’s medium under a stereomicroscope and allowed to air-dry for 2–3 days. Each slide was labeled with the corresponding species based on morphological identification, with molecular data providing complementary support. The wings were photographed using a digital camera attached to a Nikon SMZ18 stereomicroscope (Nikon Corporation, Tokyo, Japan), with a 1-mm scale bar included in each image. Overall, 180 wing images, with 45 images per species, were used for both landmark- and outline-based GM analyses (Table 1). All GM procedures were performed using XYOM (XY Online Morphometrics) version 3 (Dujardin and Dujardin, 2019), available at https://xyom.io/.

To assess the precision of landmark and outline digitization, a subset of 10 wing images from each species was randomly selected for repeated measurements. Each image was digitized twice by the same observer to evaluate intra-observer repeatability, while the same images were independently digitized by a second observer to assess inter-observer repeatability. Measurement error and repeatability were assessed using Procrustes ANOVA implemented in XYOM, as described previously (Klingenberg and McIntyre, 1998). The analysis partitioned shape variation into between-individual variance and within-individual variance associated with repeated digitization, from which repeatability was calculated.

### 2.6 Landmark-based geometric morphometric analysis

Overall, 21 homologous landmarks on the left wing of each specimen were digitized to characterize wing morphology (Fig. 5A) using the same 21-landmark configuration previously applied to *Tabanus* species by Changbunjong et al. (2025). Raw landmark coordinates are presented in Supplementary Table S1. Generalized Procrustes analysis was performed to superimpose the landmark configurations and remove variation attributable to position, orientation, and scale. Centroid size (CS), calculated as the square root of the summed squared distances of all landmarks from the centroid, was used as a measure of wing size (Bookstein, 1991). Differences in wing CS among species were evaluated using non-parametric permutation tests with 1000 permutations. Statistical significance was set at *P* < 0.05.

**Fig. 5:**
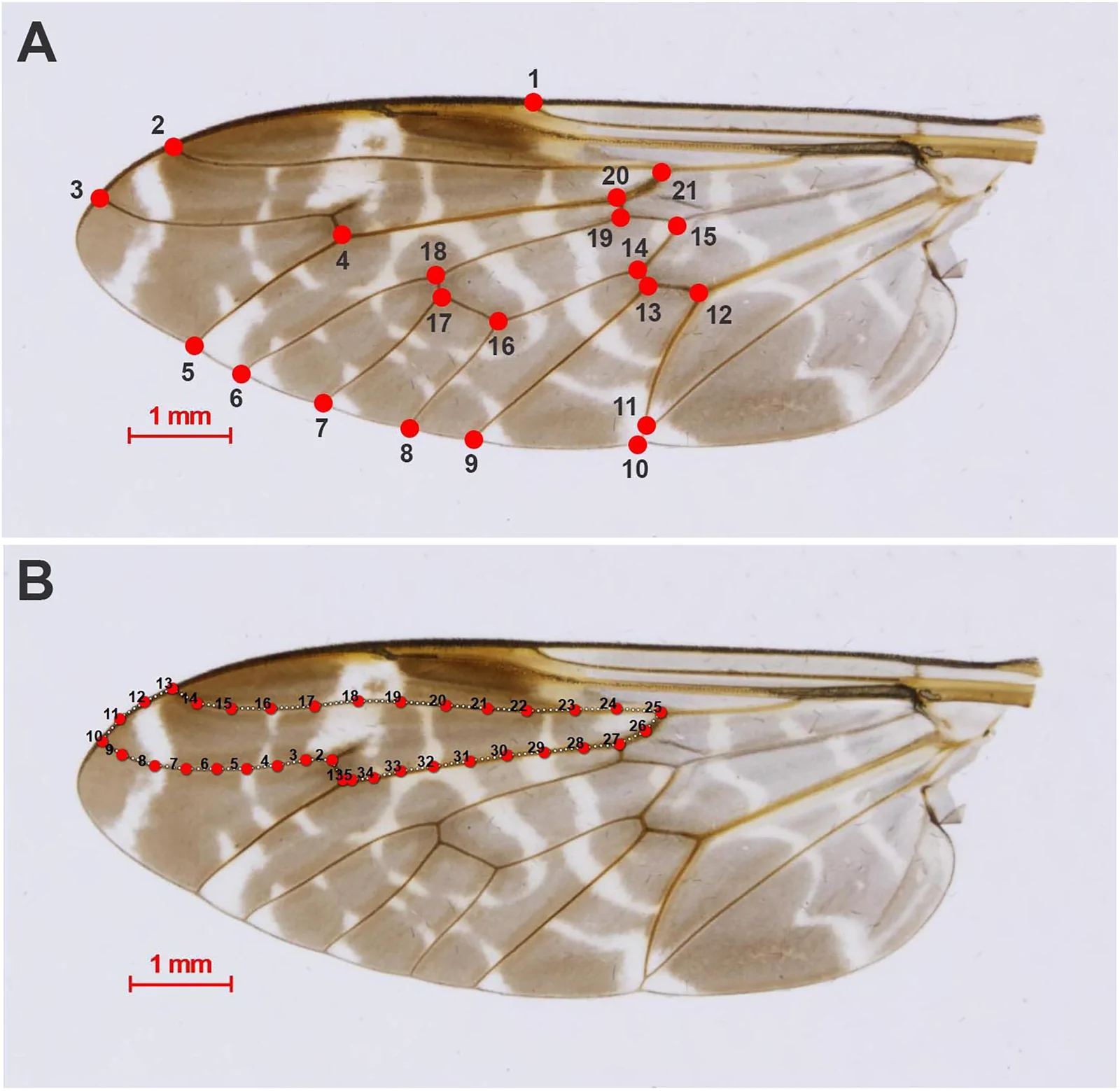
Wing configurations used for geometric morphometric analyses of *Haematopota* species. **A** Twenty-one homologous landmarks used for landmark-based analysis. **B** Outline of the first submarginal cell used for outline-based analysis.

Principal components analysis (PCA) was performed using the Procrustes shape coordinates to summarize variations in wing shape. The first 38 principal components (PCs) were retained as shape variables for subsequent analyses. Subsequently, discriminant analysis (DA) was performed using the retained PCs to evaluate inter-species wing shape differentiation. Species separation was visualized using factor maps based on the first two discriminant factors (DFs). Mahalanobis distances between species were calculated to quantify the degree of shape divergence, and their significance was assessed using non-parametric permutation tests with 1000 permutations, followed by Bonferroni correction for multiple comparisons. Statistical significance was set at *P* < 0.05.

### 2.7 Outline-based geometric morphometric analysis

For the outline-based GM approach, the contour of the first submarginal cell (Fig. 5B) was selected based on the wing-cell region previously analyzed in *Tabanus* species by Changbunjong et al. (2024) and subsequently analyzed using elliptic Fourier analysis (EFA) (Kuhl and Giardina, 1982). The raw outline coordinate data are listed in Supplementary Table S2. The perimeter of the first submarginal cell was used as a measure of contour size. Differences in contour size among species were assessed using non-parametric permutation tests with 1000 permutations. Statistical significance was set at *P* < 0.05.

Normalized elliptic Fourier coefficients (NEF) derived from EFA were used to represent the shape of the first submarginal cell. PCA was performed on the normalized Fourier coefficients, and the first 41 PCs were retained as shape variables for subsequent analyses. Discriminant analysis was performed using these PCs to assess shape differentiation among species. Species separation was visualized using factor maps based on the first two DFs. Mahalanobis distances among species were calculated to quantify pairwise differences in contour shape, with statistical significance evaluated using non-parametric permutation tests with 1000 permutations followed by Bonferroni correction. Statistical significance was set at *P* < 0.05.

### 2.8 Validated classification

The discriminatory performance of the landmark- and outline-based GM approaches was evaluated using leave-one-out cross-validation. For each iteration, one specimen was excluded from the dataset and subsequently classified using the remaining specimens as the reference dataset. Size-based classification was performed using ML (Dujardin et al., 2017), whereas shape-based classification was performed using Mahalanobis distances (Manly, 2004). Classification performance was summarized as the number and percentage of correctly classified specimens. Overall classification accuracy was calculated for each GM approach. Furthermore, the adjusted total accuracy (TAU) was calculated to account for the probability of correct assignment occurring by chance (Kovarovic et al., 2011).

### 2.9 Allometric analysis

Allometric analysis was performed to evaluate the relationship between size and shape variation in both landmark- and outline-based GM datasets. For the landmark-based dataset, PC1 of the Procrustes shape variables was regressed against CS. For the outline-based dataset, PC1 of the normalized elliptic Fourier shape variables was regressed against the semi-major axis of the first ellipse, which served as the size variable for the allometric analysis. The strength of the size-shape relationships was assessed using Pearson’s correlation coefficient (*r*), while the proportion of shape variation associated with size was quantified using the coefficient of determination (*R*^2^).

## 3 Results

Overall, 180 wing images representing *H. javana*, *H. libera*, *H. pachycera*, and *H. varifrons* were included in the landmark- and outline-based GM analyses, with 45 specimens per species. Molecular analysis was also performed to confirm the taxonomic identities of representative specimens using *cox*1 DNA barcoding.

### 3.1 Molecular confirmation of species identity

In total, 15 *cox*1 sequences were successfully obtained from three specimens of each of the five morphologically identified *Haematopota* species (*H. javana*, *H. libera*, *H. pachycera*, *H. singularis*, and *H. varifrons*). BLAST searches against GenBank and Barcode Identification Engine searches in BOLD consistently confirmed the morphological identification of the four species; GenBank matches for *H. javana* showed 96.66–100% similarity across four reference sequences, whereas BOLD matches showed 96.01–100% similarity across nine sequences. The three *H. libera* sequences showed 99.24% similarity to the available reference sequence in both GenBank and BOLD. The three *H. pachycera* sequences showed 94.22–98.94% similarity to five GenBank reference sequences and 94.18–100% similarity to nine BOLD reference sequences. The three *H. singularis* sequences showed 99.70% similarity to two GenBank sequences and 99.69–99.70% similarity to three BOLD sequences (Table 2).

**Table 2:** Results of *cox*1 DNA barcoding for *Haematopota* spp. based on GenBank and BOLD database comparisons.

ID	Species	GenBank ID	GenBank		BOLD	
			Species match	% Similarity (*n*)	Species match	% Similarity (*n*)
1	*H. javana*	PZ761172	*H. javana*	96.66–100 (4)	*H. javana*	96.01–100 (9)
2	*H. javana*	PZ761173	*H. javana*	96.66–100 (4)	*H. javana*	96.01–100 (9)
3	*H. javana*	PZ761174	*H. javana*	96.66–100 (4)	*H. javana*	96.01–100 (9)
4	*H. libera*	PZ761175	*H. libera*	99.24 (1)	*H. libera*	99.24 (1)
5	*H. libera*	PZ761176	*H. libera*	99.24 (1)	*H. libera*	99.24 (1)
6	*H. libera*	PZ761177	*H. libera*	99.24 (1)	*H. libera*	99.24 (1)
7	*H. pachycera*	PZ761178	*H. pachycera*	94.22–98.63 (5)	*H. pachycera*	94.18–99.51 (9)
8	*H. pachycera*	PZ761179	*H. pachycera*	94.68–98.94 (5)	*H. pachycera*	94.64–100 (9)
9	*H. pachycera*	PZ761180	*H. pachycera*	94.68–98.94 (5)	*H. pachycera*	94.64–100 (9)
10	*H. singularis*	PZ761181	*H. singularis*	99.70 (2)	*H. singularis*	99.69–99.70 (3)
11	*H. singularis*	PZ761182	*H. singularis*	99.70 (2)	*H. singularis*	99.69–99.70 (3)
12	*H. singularis*	PZ761183	*H. singularis*	99.70 (2)	*H. singularis*	99.69–99.70 (3)
13	*H. varifrons*	PZ761184	No match	No match	No match	No match
14	*H. varifrons*	PZ761185	No match	No match	No match	No match
15	*H. varifrons*	PZ761186	No match	No match	No match	No match

*Note*: All comparisons had 100% query coverage, indicating that the full query sequence was aligned with the reference sequence. No match indicates that no suitable sequence match was found in the respective database. *Abbreviations*: *n*, number of sequences.

No matching sequences were obtained from either GenBank or BOLD for the three *H. varifrons* sequences. Nevertheless, the morphologically identified *H. varifrons* specimens comprised a distinct and well-supported clade in the phylogenetic analysis. The ML tree grouped the five morphologically identified *Haematopota* species into distinct clades, with bootstrap support of 96–100% (Fig. 6). The absence of database matches for *H. varifrons* highlighted the paucity of publicly accessible reference sequences for this species.

**Fig. 6:**
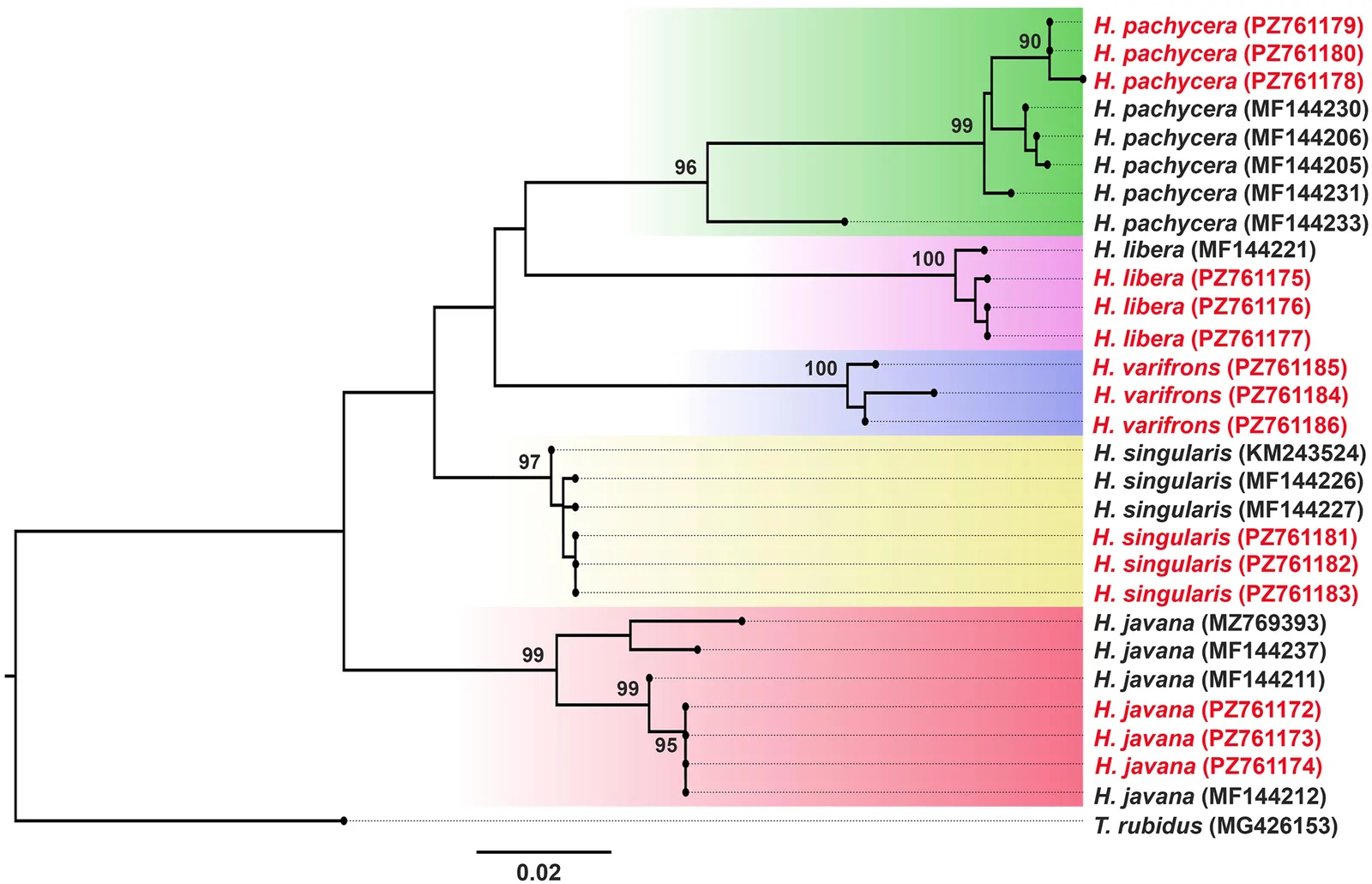
Maximum-likelihood phylogenetic tree based on mitochondrial *cox*1 sequences of five *Haematopota* species, including 15 sequences generated in this study (*red*) and 13 reference sequences retrieved from GenBank (*black*). *Tabanus rubidus* was used as the outgroup. Bootstrap values ≥ 90% are indicated on the corresponding branches.

### 3.2 Measurement precision and repeatability

Procrustes ANOVA showed high measurement precision for both landmark- and outline-based GM analyses. The intra- and inter-observer repeatability rates were both 98% for both methods, indicating high consistency in repeated measurements by the same observer and between observers. Furthermore, only a small proportion of variation was attributable to measurement error.

### 3.3 Wing and wing-cell size variation

Significant size differences were observed among the four *Haematopota* species for both landmark- and outline-based measurements. The distributions of CS from landmark-based GM and perimeter from outline-based GM are depicted in box plots (Fig. 7A and B, respectively). Based on CS, *H. pachycera* had the largest mean wing size (8.81 mm), followed by *H. javana* (7.55 mm), *H. varifrons* (7.17 mm), and *H. libera* (6.19 mm). The mean CS of *H. javana* and *H. varifrons* did not differ significantly, whereas the other species pairs showed significant differences (Table 3).

**Fig. 7:**
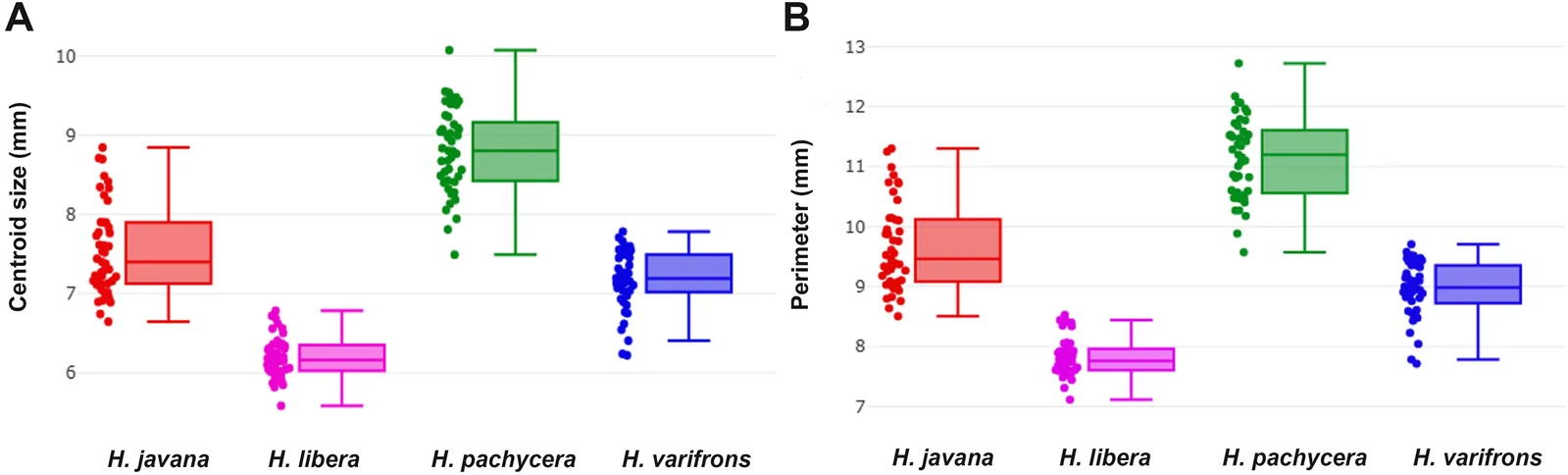
Boxplots illustrating size variation among four *Haematopota* species. **A** Wing size based on centroid size from landmark-based geometric morphometric (GM) analysis. **B** First submarginal cell size based on perimeter from outline-based GM analysis. The horizontal line within each box represents the median; the lower and upper boundaries represent the 25th and 75th percentiles, respectively. Colored dots represent individual specimens.

**Table 3:** Wing size variation among four *Haematopota* species based on centroid size and perimeter of the first submarginal cell.

Species	*n*	Mean (mm)	Range	Variance	SD
**Centroid size**					
*H. javana*	45	7.55^A^	6.65–8.84	0.32	0.57
*H. libera*	45	6.19^B^	5.58–6.79	0.07	0.26
*H. pachycera*	45	8.81^C^	7.49–10.07	0.29	0.54
*H. varifrons*	45	7.17^A^	6.22–7.78	0.14	0.37
**Perimeter**					
*H. javana*	45	9.66^A^	8.51–11.30	0.53	0.73
*H. libera*	45	7.83^B^	7.11–8.53	0.10	0.32
*H. pachycera*	45	11.14^C^	9.57–12.72	0.46	0.68
*H. varifrons*	45	8.96^D^	7.72–9.70	0.21	0.46

*Note*: Different superscript letters indicate statistically significant differences (*P* < 0.05) among species within each size measure.

*Abbreviations*: *n*, number of specimens; SD, standard deviation.

A similar pattern was observed for the perimeter of the first submarginal cell contour. *Haematopota pachycera* had the largest mean perimeter (11.14 mm), followed by *H. javana* (9.66 mm), *H. varifrons* (8.96 mm), and *H. libera* (7.83 mm). All pairwise comparisons differed significantly (Table 3).

### 3.4 Wing and wing-cell shape variation

Landmark-based GM analysis using 21 homologous landmarks revealed differences in wing shape among the four *Haematopota* species. The mean shape configurations showed generally similar overall wing shapes among the four species, with some differences in the positions of several landmarks (Fig. 8A). DA revealed relatively clear separation of the four species groups, although slight overlap was observed between *H. libera* and *H. pachycera* in the factor map. DF1 and DF2 accounted for 46.7% and 31.4% of the total discriminant variation, respectively (Fig. 8B). Despite this overlap, pairwise Mahalanobis distances revealed significant differences in wing shape between all species pairs after Bonferroni correction (*P* < 0.05; Table 4), indicating significant interspecific differentiation in multivariate wing shape.

**Fig. 8:**
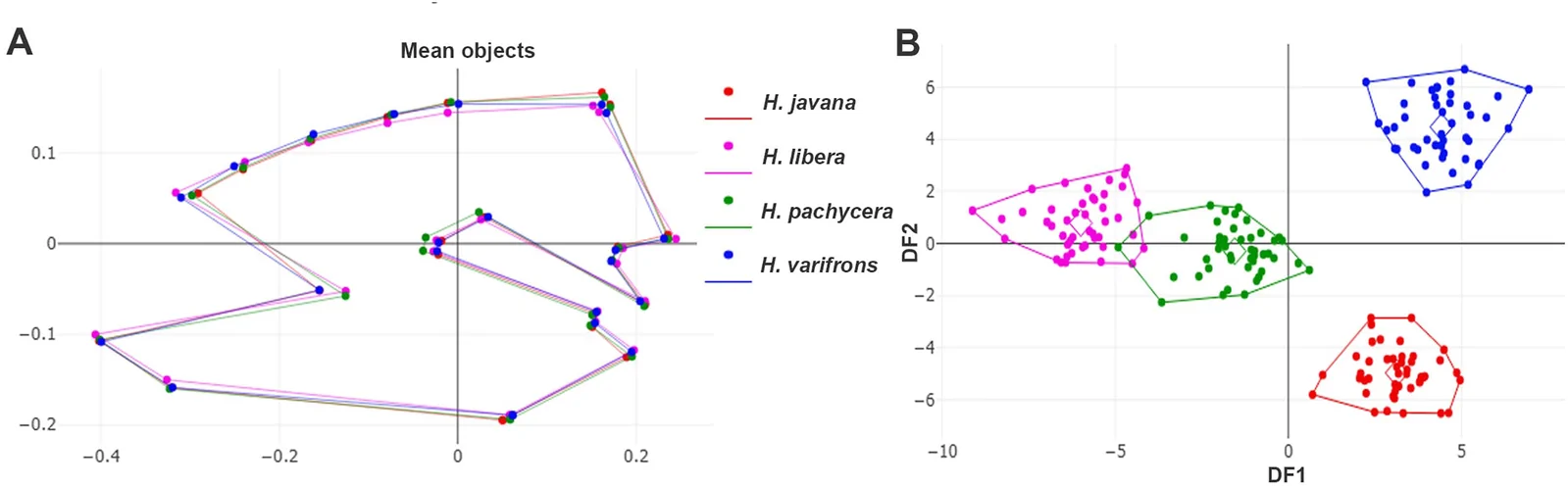
**A** Superimposed mean configurations of the 21 wing landmarks for four *Haematopota* species. **B** Factor map of the first two discriminant factors (DFs) from landmark-based geometric morphometric analysis. Points indicate individual specimens. DF1 and DF2 accounted for 46.7% and 31.4% of the total discriminant variation, respectively.

**Table 4:** Pairwise Mahalanobis distances among four *Haematopota* species based on landmark- and outline-based geometric morphometric analyses.

GM approach	Species	*H. javana*	*H. libera*	*H. pachycera*
Landmark-based GM	*H. javana*	–		
	*H. libera*	10.85	–	
	*H. pachycera*	9.03	8.61	–
	*H. varifrons*	9.56	11.19	9.44
Outline-based GM	*H. javana*	–		
	*H. libera*	8.99	–	
	*H. pachycera*	9.19	7.04	–
	*H. varifrons*	6.51	9.36	10.97

*Note*: All pairwise Mahalanobis distances were significant after Bonferroni correction (*P* < 0.05).

Outline-based GM analysis of the first submarginal cell contour also revealed shape differences among the four *Haematopota* species. The mean superimposed outlines revealed differences in the shape of the first submarginal cell, with *H. javana* and *H. varifrons* showing relatively similar mean outlines, while *H. libera* and *H. pachycera* exhibited more similar outlines (Fig. 9A). DA identified distinct and well-separated species groups with no overlap in the factor map. DF1 and DF2 accounted for 62.5% and 24.3% of the total discriminant variation, respectively (Fig. 9B). Pairwise Mahalanobis distances were significant for all species comparisons after Bonferroni correction (*P* < 0.05; Table 4), indicating significant differentiation in the shape of the first submarginal cell contour among all four species.

**Fig. 9:**
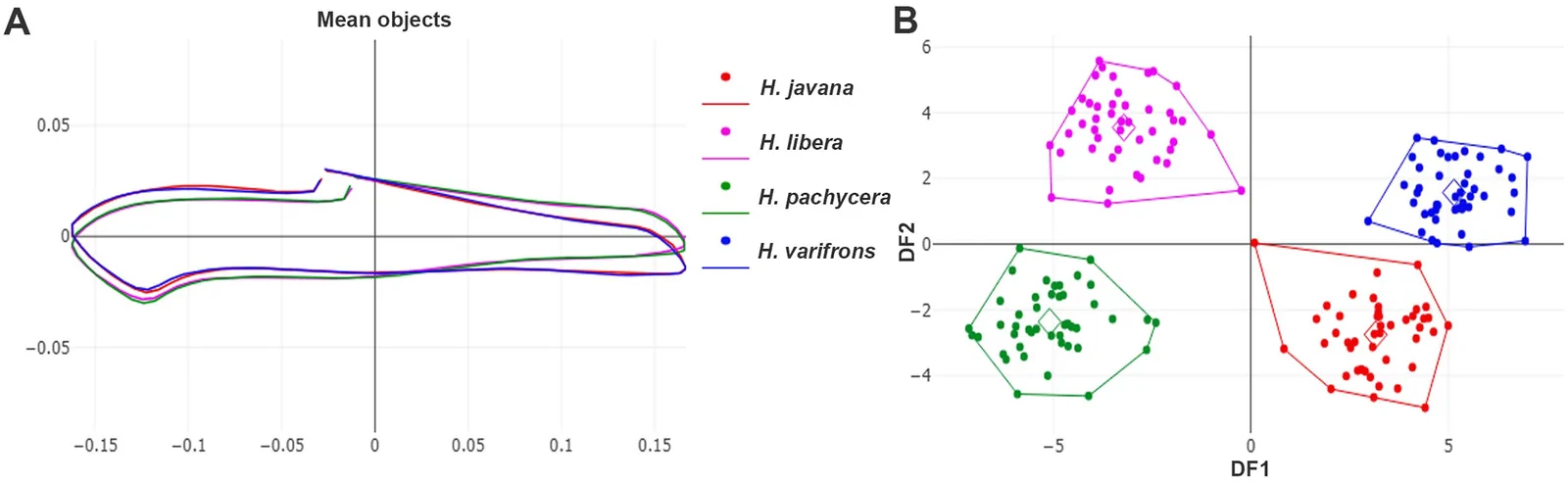
**A** Superimposed mean contours of the first submarginal cell for four *Haematopota* species. **B** Factor map of the first two discriminant factors (DFs) from outline-based geometric morphometric analysis. Points represent individual specimens. DF1 and DF2 accounted for 62.5% and 24.3% of the total discriminant variation, respectively.

### 3.5 Validated classification

Leave-one-out cross-validation demonstrated variations in classification performance between size and shape for both GM approaches. For the landmark-based approach, wing size correctly classified 122 of 180 specimens (67.78%), whereas wing shape correctly classified all 180 specimens (100%). Species-specific size-based classification accuracy ranged from 35.56% in *H. javana* to 91.11% in *H. libera*, while shape-based classification was 100% for all four species. For the outline-based approach, perimeter-based size classification correctly assigned 121 of 180 specimens (67.22%), with species-specific accuracy ranging from 44.44% for *H. javana* to 93.33% for *H. libera*. Classification based on the contour shape of the first submarginal cell correctly assigned 176 of 180 specimens (97.78%), with a classification accuracy of 97.78% for each of the four species. The TAU further indicated substantially higher classification performance for shape than size. For the landmark-based approach, TAU was 100% for wing shape compared with 57% for wing size. For the outline-based approach, TAU was 97% for first submarginal cell contour shape compared with 57% for perimeter-based size classification (Table 5).

**Table 5:** Validated classification of the four *Haematopota* species based on size and shape using landmark- and outline-based geometric morphometric approaches.

Species	Size		Shape	
	Assigned	Accuracy (%)	Assigned	Accuracy (%)
**Landmark-based GM**				
*H. javana* (*n* = 45)	16	35.56	45	100
*H. libera* (*n* = 45)	41	91.11	45	100
*H. pachycera* (*n* = 45)	39	86.67	45	100
*H. varifrons* (*n* = 45)	26	57.78	45	100
Total accuracy (*n* = 180)	122	67.78	180	100
Adjusted total accuracy		57.00		100
**Outline-based GM**				
*H. javana* (*n* = 45)	20	44.44	44	97.78
*H. libera* (*n* = 45)	42	93.33	44	97.78
*H. pachycera* (*n* = 45)	36	80.00	44	97.78
*H. varifrons* (*n* = 45)	23	51.11	44	97.78
Total accuracy (*n* = 180)	121	67.22	176	97.78
Adjusted total accuracy		57.00		97.00

*Note*: For landmark-based GM, size and shape refer to whole-wing centroid size and wing shape, respectively. For outline-based GM, size and shape refer to the perimeter and contour shape of the first submarginal cell, respectively.

*Abbreviations*: *n*, number of specimens.

### 3.6 Allometric effect

Both GM approaches revealed a positive relationship between size and shape. For the landmark-based analysis (Fig. 10A), PC1 of wing shape was positively correlated with CS (*r* = 0.39), with size explaining 15.0% of the shape variation. For the outline-based analysis (Fig. 10B), PC1 of the first submarginal cell contour shape was positively correlated with the semi-major axis of the first ellipse (*r* = 0.38), with size accounting for 14.7% of the shape variation.

**Fig. 10:**
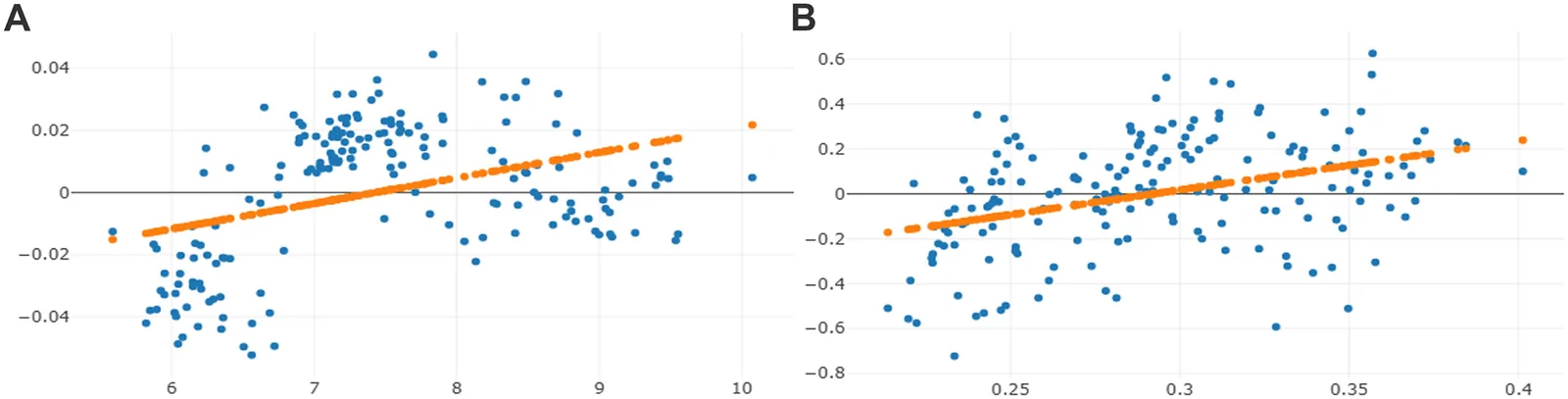
Allometric relationships between size and shape in four *Haematopota* species. **A** Relationship between centroid size and PC1 of wing shape based on landmark-based geometric morphometric analysis. **B** Relationship between the semi-major axis of the first ellipse and PC1 of first submarginal cell contour shape based on outline-based geometric morphometric analysis.

## 4 Discussion

The present study demonstrated the utility of wing morphology in providing reliable discriminatory information for distinguishing *Haematopota* species, particularly when wing shape rather than wing size is considered. Morphological species assignments were supported by *cox*1 DNA barcoding and phylogenetic analysis, providing complementary molecular evidence for the GM approaches. Although *H. singularis* was collected and molecularly characterized, it was not included in the GM analyses because the 10 adult female specimens collected were insufficient to establish a robust reference dataset for morphometric analysis. Nevertheless, the molecular characterization of the species provides useful information for species identification. The *cox*1 sequences of *H. javana*, *H. libera*, *H. pachycera*, and *H. singularis* showed high similarity to reference sequences in GenBank and BOLD. These findings are consistent with previous molecular-based identification work in Thailand showing that *cox*1 barcoding can successfully discriminate among *Haematopota* species and define distinct species-level phylogenetic groups (Nitiyamatawat et al., 2017). However, no matching reference sequences were available for *H. varifrons*. The sequences generated in the present study therefore provide additional molecular reference data for this species and help expand the barcode resources available for *Haematopota* spp. in Thailand. The relatively broad sequence similarity observed for *H. pachycera* may also be consistent with the relatively high intraspecific *cox*1 variation previously reported for this species in Thailand (Nitiyamatawat et al., 2017). This finding indicates that DNA barcoding should preferably be interpreted using multiple reference sequences, along with morphological and phylogenetic evidence, rather than relying solely on a single database match (Krčmar et al., 2022).

Both wing size and wing shape differed significantly among the four species, although wing shape provided substantially greater discriminatory power. While *H. pachycera* had the largest wings and *H. libera* had the smallest, overlap in CS between *H. javana* and *H. varifrons* indicates that wing size alone is insufficient for reliable species discrimination. However, environmental conditions during larval development may also influence wing size and geometry. In the present study, *H. javana* was collected from Thong Pha Phum District, whereas the other three species were collected from Mueang District, both in Kanchanaburi Province. These geographical differences cannot be completely excluded as potential sources of variation in wing size. This represents a limitation of the present study, as information on the larval habitats of the sampled populations was not available. A previous study on *Stomoxys calcitrans* showed that larval density and substrate quality can affect wing geometry (Baleba et al., 2019). In contrast, shape-based classification achieved 100% accuracy for the landmark-based approach and 97.78% for the outline-based approach, compared with only 67.78% and 67.22%, respectively, for size-based classification. The higher TAU values for shape indicate better chance-adjusted classification performance than size in the present dataset. These findings align with previous studies of hematophagous flies and other medically important flies, in which wing shape provided useful information for species discrimination and successfully separated morphologically similar taxa (Sontigun et al., 2019; Ardkhongharn et al., 2023; Changbunjong et al., 2025).

Although both methods performed well, the landmark-based approach showed slightly higher classification performance than the outline-based approach. Landmark-based shape correctly classified all specimens during cross-validation, whereas outline-based shape correctly classified 97.78% of the specimens. These findings corroborate previous work on *Tabanus* spp., where whole-wing landmark-based GM achieved higher classification accuracy than outline-based GM of individual wing cell contours (Changbunjong et al., 2021, 2024). This difference suggests a greater amount of morphological information captured by multiple homologous landmarks distributed across the wing compared with the contour of a single wing cell (Changbunjong et al., 2024). Nevertheless, the high accuracy of the outline-based approach indicates that the shape of the first submarginal cell also contains substantial species-specific information. The small number of misclassified specimens in the outline-based analysis suggests that some interspecific similarity remains in the shape of the first submarginal cell contour. Overall, these findings suggest that landmark-based GM may capture broader aspects of wing shape, whereas outline-based GM provides a useful alternative when homologous landmarks cannot be reliably digitized.

The high measurement repeatability observed in both GM approaches supports the reliability of the detected interspecific differences. The relatively small contribution of measurement error indicates that the observed separation among species was unlikely to be substantially affected by digitization variability. Both approaches also revealed a positive association between size and shape, with size accounting for approximately 15% of shape variation. This indicates that allometry contributed to variation in both wing shape and first submarginal cell contour shape but did not account for most of the observed differences, supporting the interpretation that species-specific shape variation was an important component in discrimination. Similar relationships between size and wing shape have been reported in other insect GM studies, emphasizing the need to consider allometry when interpreting interspecific shape variation (Klingenberg, 2016; Sontigun et al., 2017; Laojun et al., 2023).

Overall, the findings indicate that wing shape, particularly when characterized using landmark-based GM, provides useful discriminatory information for the sampled species of *Haematopota*. DNA barcoding provided complementary molecular evidence in the present study but was limited by the availability of suitable reference sequences. Wing GM may therefore complement conventional morphological examination by providing quantitative information on wing shape for species discrimination. The complementary advantages of molecular data and wing GM may be particularly useful for investigating *Haematopota* species that are morphologically similar or poorly represented in molecular databases. Recent advances in deep learning-based image analysis have also enabled automated landmark and semilandmark detection in Diptera wing images, which could further improve the efficiency and reproducibility of wing GM analyses (Geldenhuys et al., 2023; Nolte et al., 2026). Future studies should expand sampling to additional locations and populations, increase molecular sampling, and evaluate larger independent datasets to further assess the applicability and robustness of wing GM for species discrimination in *Haematopota*. Automated landmark detection could also be explored to facilitate more efficient and reproducible GM analyses in future studies.

## 5 Conclusions

This study demonstrated that wing GM effectively discriminated among the sampled *Haematopota* species. Wing shape showed substantially greater discriminatory power than wing size, with landmark-based GM achieving 100% classification accuracy. Outline-based GM also demonstrated high performance, indicating its potential as an alternative method for specimens with damaged wings but intact first submarginal cell contour. The complementary use of landmark- and outline-based approaches may therefore enhance the flexibility of wing GM for species discrimination in *Haematopota*. Further studies with broader geographical sampling and larger datasets from independent populations are warranted to evaluate the generalizability and robustness of these approaches.

## Ethical approval

The study protocol was approved by the Animal Care and Use Committee of the Faculty of Veterinary Science, Mahidol University, Thailand (Ref. No. MUVS-2025-05-24).

## CRediT authorship contribution statement

**Pichayapa Som-udorn:** Writing – original draft, Methodology, Investigation, Formal analysis. **Pattarakorn Khongtaweboon:** Writing – original draft, Methodology, Investigation, Formal analysis. **Ayarin Wijitthavornkun:** Writing – original draft, Methodology, Investigation, Formal analysis. **Sivapong Sungpradit:** Writing – original draft, Visualization, Methodology, Investigation, Conceptualization. **Tanawat Chaiphongpachara:** Writing – review & editing, Writing – original draft, Visualization, Methodology, Formal analysis, Data curation, Conceptualization. **Tanasak Changbunjong:** Writing – review & editing, Writing – original draft, Visualization, Supervision, Project administration, Methodology, Investigation, Funding acquisition, Formal analysis, Data curation, Conceptualization.

## Statement on the use of AI-assisted technologies

During the preparation of this work the authors used ChatGPT-5.6 to assist with checking grammar, sentence structure, and language clarity. All content was subsequently reviewed and edited by the authors, who take full responsibility for the final version of the publication.

## Funding

This work was supported by the 10.13039/501100020619Faculty of Veterinary Science, 10.13039/501100004156Mahidol University, Thailand (Grant No. 3/2569).

## Declaration of competing interest

The authors declare that they have no known competing financial interests or personal relationships that could have appeared to influence the work reported in this paper.

## Data Availability

The data supporting the conclusions of this article are included in the article and its supplementary materials. The newly generated sequences were submitted to the GenBank database under the accession numbers PZ761172-PZ761186. The wing images used in this study are openly available on Figshare at https://doi.org/10.6084/m9.figshare.33991912.
